# A Randomized Trial to Compare the Safety, Tolerability, and Effectiveness of 3 Antimalarial Regimens for the Prevention of Malaria in Nigerian Patients With Sickle Cell Disease

**DOI:** 10.1093/infdis/jiv093

**Published:** 2015-02-20

**Authors:** Rasaq Olaosebikan, Kolade Ernest, Kalifa Bojang, Olugbenga Mokuolu, Andrea M. Rehman, Muna Affara, Davis Nwakanma, Jean-René Kiechel, Taofik Ogunkunle, Tope Olagunju, Rukayat Murtala, Peter Omefe, Tosin Lambe, Surajudeen Bello, Olayinka Ibrahim, Benedict Olorunsola, Ayotade Ojuawo, Brian Greenwood, Paul Milligan

**Affiliations:** 1 University of Ilorin Teaching Hospital, Nigeria; 2 Medical Research Council Unit, Banjul, Gambia; 3 University of Nebraska Medical Centre, Omaha; 4 Drugs for Neglected Diseases Initiative, Geneva, Switzerland; 5 London School of Hygiene and Tropical Medicine, United Kingdom

**Keywords:** sickle cell disease, malaria, chemoprevention, intermittent preventive treatment, prophylaxis, mefloquine-artesunate, proguanil

## Abstract

**Background:**

Malaria prophylaxis is recommended for persons with sickle cell disease (SCD), but the value of this has been questioned. The aim of this study was to find out whether intermittent preventive treatment (IPT) with a fixed-dose combination of mefloquine-artesunate (MQAS) or sulfadoxine-pyrimethamine plus amodiaquine (SPAQ) was more effective than daily proguanil for malaria prevention in subjects with SCD.

**Methods:**

Patients with SCD were randomized to receive daily treatment with proguanil or IPT with either MQAS or SPAQ once every 2 months at routine clinic visits. Patients were followed up for 14 months.

**Findings:**

A total of 270 patients with SCD were studied, with 90 in each group. Adherence to the IPT regimens was excellent, but 57% of patients took <75% of their daily doses of proguanil. IPT was well tolerated; the most common side effects were vomiting and abdominal pain. Protective efficacy against malaria, compared with daily proguanil, was 61% (95% confidence interval, 3%–84%) for MQAS and 36% (40%–70%) for SPAQ. There were fewer outpatient illness episodes in children who received IPT than those who received proguanil.

**Conclusions:**

IPT with MQAS administered to patients with SCD during routine clinic visits was well tolerated and more effective in preventing malaria than daily prophylaxis with proguanil.

**Clinical Trials Registration:**

NCT01319448 and ISRCTN46158146.

Although the incidence of *Plasmodium falciparum* infection is thought to be lower in children with sickle cell disease (SCD) than in normal children [1], the consequence of malaria in SCD can be serious. Findings of studies in Kenya [2] and Tanzania [3] in which the confounding effects of prophylaxis for patients with SCD could be excluded were consistent with a reduced prevalence of *P. falciparum* parasitemia and incidence of *P. falciparum* infection in patients with HbSS, compared with children with HbAA. However, in the Kenyan study, mortality in children with SCD hospitalized with malaria was substantially higher than that among children without SCD [3]. Malaria can aggravate severe anemia and like, other infections, can lead to crises in children with SCD, and the complications of a crisis precipitated by *P. falciparum* infection can be fatal [4, 5]. Malaria prevention in SCD has, therefore, been considered to be essential in regions where malaria is endemic, but the evidence base for current drug policies, which rely mainly on daily proguanil therapy or weekly pyrimethamine or chloroquine therapy, is extremely weak. A Cochrane review of malaria prophylaxis for patients with SCD concluded that prophylaxis is beneficial, with fewer episodes of malaria, higher mean hemoglobin concentrations, fewer transfusions, fewer hospital admissions, and fewer sickle cell crises in patients receiving prophylaxis [6], but the review included only 2 small studies, one from Kampala in 1965, which involved administration of chloroquine plus benzathine penicillin for 12 months [7], and one from Nigeria in 2003, in which 97 patients were randomized to receive daily proguanil therapy, weekly pyrimethamine therapy, or placebo for 9 months [8]. Studies not in the review included a placebo-controlled trial of 60 patients in Senegal that showed that monthly sulfadoxine-pyrimethamine (SP) treatment during the transmission season was well tolerated and reduced morbidity [9]; a study in Uganda, with just 1 month of follow-up, that suggested that weekly chloroquine therapy was less effective than monthly SP therapy [10]; and a study in Nigeria that indicated that weekly mefloquine (MQ) was well tolerated and more effective than daily proguanil [11]. Several small surveys of patients with SCD in Nigeria who received daily proguanil or weekly pyrimethamine prophylaxis have found that malaria parasitemia is common and attributed this finding to poor compliance and resistance [12–14]. The prevalence of mutations in the genes encoding dihydrofolate reductase (*dhfr*) and dihydropteroate synthase (*dhps*) has been little studied but appears to be high [15].

Adherence to a daily or weekly prophylactic regimen is likely to be poor and difficult to sustain over long periods. The national antimalarial policy in Nigeria for protection of patients with SCD against malaria at the time that this trial was conducted was daily proguanil, and proguanil prophylaxis continues to be used widely in Nigeria, although more-recent guidelines (from 2011) state that, because proguanil may not be effective, patients with SCD should rely on long-lasting insecticide-treated bed nets and, if they have malaria, prompt treatment.

Chemoprevention with drugs being given under supervision by health workers is increasingly being used to prevent malaria in countries of endemicity. The World Health Organization now recommends intermittent preventive treatment (IPT) with SP for prevention of malaria in pregnant women, administered during antenatal clinic visits; IPT with SP in infants (IPTi), given when children come for routine vaccinations; and, in areas of seasonal transmission, seasonal malaria chemoprevention with SP plus amodiaquine (SPAQ) for children <5 years of age, delivered monthly at home by a community health worker. Patients with SCD who are stable are recommended to visit the clinic regularly, providing an opportunity to administer chemoprevention under supervision, but for this approach to be effective, a long-acting antimalarial drug combination is needed.

SPAQ is effective when administered monthly, but protection wanes rapidly after about 4 weeks [16]. MQ-artesunate (MQAS), given over 3 days, is highly effective in treating uncomplicated malaria. A fixed dose of MQAS developed by the Drugs for Neglected Diseases Initiative is better tolerated, with a lower incidence of vomiting, than the separate-tablet regimen [17]. When the duration of protection provided by different IPTi drug regimens given at 2, 3, and 9 months of age was estimated, MQ gave the longest period of protection, with an estimated efficacy of 73% in the first month and 73% in the second month after treatment [18].

In Nigeria, patients with SCD who are stable are recommended to attend the clinic once every 2 months. The aim of this trial was, therefore, to compare the efficacy, safety, and tolerability of either IPT with MQAS given over 3 days or SPAQ (a single dose of SP plus AQ over 3 days) administered under supervision at routine bimonthly (once every 2 months) clinic visits to the efficacy, safety, and tolerability of the standard regimen of daily proguanil in patients with SCD.

## METHODS

### Study Design

#### Study Population

This study was undertaken at the SCD clinic of the Outpatient Unit of the University of Ilorin Teaching Hospital, Kwara state, Nigeria. Patients aged ≥6 months and weighing ≥5 kg with a documented genotype of SS or SC were invited to participate. Those who had an acute illness or any additional chronic disease, had a known allergy to any of the study drugs, or had been treated during the 2 weeks prior to recruitment with SP, AQ, or MQ were excluded. Signed consent was sought from the participants or their parents after the aims and procedures of the study had been explained.

#### Randomization

A list of randomly permuted blocks of 9, generated using Stata, version 11.1, was used to randomly assign eligible patients to receive bimonthly IPT with MQAS or SPAQ or standard daily treatment with proguanil. Participants were asked to return to the clinic once every 2 months for the next year to receive their treatment and, in the case of the proguanil group, to be given the next supply of proguanil tablets. Treatment allocations were kept inside opaque sealed envelopes. When a participant was enrolled, they were assigned the next envelope in numerical sequence. Their name and the date were written on the envelope, which was then opened to determine treatment allocation. Participants were issued identification cards bearing the participant's name, study number, and phone numbers of the investigators and were contacted by phone the day before each clinic visit to remind them to attend.

#### Procedures at Each Scheduled Clinic Visit

Participants were asked to record any illness in a diary, and at each clinic visit patients were asked about symptoms experienced since their last visit. They were then given a clinical examination, a venous blood sample was collected for hematological and biochemical measurements, a finger-prick blood sample was obtained for making thick and thin blood smears for microscopy, and a blood spot was collected onto filter paper for polymerase chain reaction (PCR) detection of *P. falciparum* and analysis of molecular markers of drug resistance. Nested PCR was used to amplify fragments at loci of 3 genes in which mutations are associated with pyrimethamine resistance (codons 51, 59, 108, and 164 of *dhfr*), sulfadoxine resistance (codons 436, 437, 540, and 581 of *dhps*), and chloroquine resistance (codon 81 of *mdr1*, which encodes multidrug resistance gene 1) [19, 20].

#### Drug Administration

For patients assigned to receive bimonthly IPT, the first dose of the 3-day regimen was administered by study staff, and the remaining doses were given to the participant or their caregiver to administer at home on each of the next 2 days. For those in the proguanil group, a dose of proguanil was administered, unused tablets were counted, and a supply of tablets for the next 2 months was given to the participant or their caregiver. All patients were given folic acid tablets and vitamin C tablets, to be taken daily, the standard of care for SCD in Nigeria. Study participants were asked to return 3 days after the clinic visit to be interviewed about adverse events over the previous 3 days and, for those in the IPT groups, to check adherence to the home doses by tablet counts. Those who were not able to be present for interview were contacted by phone, and details were cross-checked at their next visit. Participants were asked to come to the hospital if they were unwell at any time, where they were managed as outpatients or admitted to the pediatric ward. If a patient had suspected malaria, a rapid diagnostic test was performed to determine treatment.

The study was open label; although participants and study physicians unblinded to treatment, laboratory staff were unaware of treatment allocations. Laboratory methods are described in the Supplementary Materials. The primary end point, for which the trial was powered, was the occurrence of any adverse event. Secondary end points included the occurrence of vomiting, adherence to the regimen, the incidence of malaria, the incidence of illnesses treated in outpatient settings, and the incidence of illnesses treated in inpatient settings. Statistical analysis was based on intention to treat and included all patients who were randomized. Statistical methods are described in the Supplementary Materials.

### Malaria Case Management

Any study subject who had malaria (defined on the basis of an axillary temperature of ≥37.5°C or a history of fever or vomiting within the last 24 hours, together with a positive results of a malaria rapid diagnostic test) was treated with artemether/lumefantrine over 3 days. If this malaria treatment occurred at a scheduled clinic visit, the patient was not given the trial medications for that visit.

### Ethics Approvals

Ethical approval for the study was obtained from the ethics committee of the University of Ilorin Teaching Hospital and the London School of Hygiene and Tropical Medicine. An independent data safety monitoring board provided oversight for the trial.

## RESULTS

### Characteristics of the Study Participants at Enrollment

**Figure 1.**
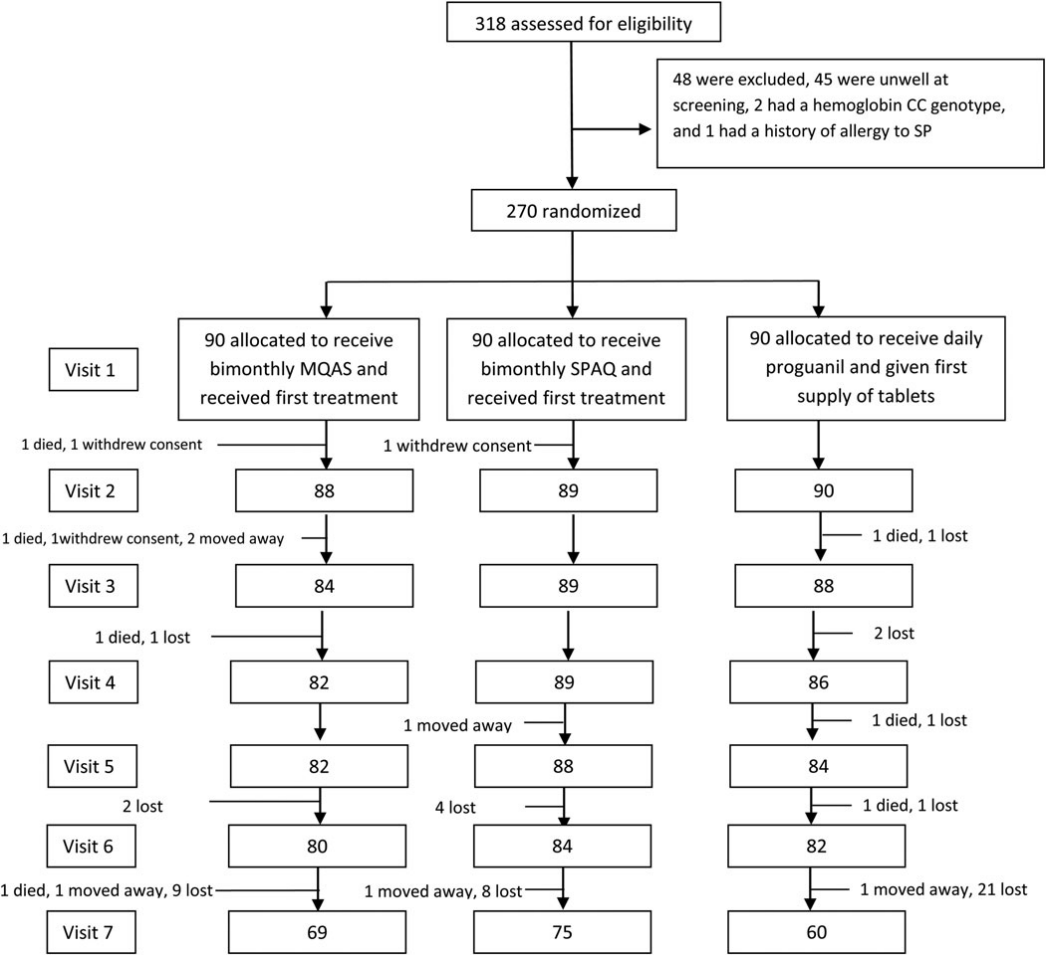
Trial profile. Each visit represents a bimonthly visit to the sickle cell clinic. Abbreviations: MQAS, mefloquine-artesunate; SP, sulfadoxine-pyrimethamine; SPAQ, sulfadoxine-pyrimethamine plus amodiaquine.

Patients were recruited from 26 September 2011 to 12 April 2012, and follow-up for the last patients ended in April 2013. A total of 318 patients were screened; 270 who met inclusion criteria were randomly assigned in a 1:1:1 ratio to receive MQAS once every 2 months, SPAQ once every 2 months, or daily proguanil. Baseline characteristics were similar in the 3 groups (Table 1 and Supplementary Table 1). Twenty-three of 270 samples (9%) were positive for *P. falciparum* by PCR at baseline, of which 9 (39%) carried the *mdr1* mutation N86Y, 20 (87%) carried the *dhfr* triple mutations S108N, N51I, and C59R, and 18 (78%) carried the *dhps* double mutations A437G and K540E. The flow of participants through the study is shown in Figure 1.

**Table 1.** Baseline Characteristics of Study Participants, by Prophylaxis Group

Variable	MQAS Group (n = 90)	SPAQ Group (n = 90)	Proguanil Group (n = 90)
Female sex	45	41	43
Age, y			
<5	30	29	24
5–9	28	28	31
10–14	18	21	21
15–25	14	12	14
Hb genotype			
SC	1 (1.1)	5 (5.6)	6 (6.7)
SS	89 (99)	85 (94)	84 (93)
Hb concentration, g/dL, mean (SD)	7.4 (1.3)	7.5 (1.6)	7.5 (1.7)
PCV, %, mean (SD)	24.6 (3.7)	25.1 (3.9)	24.6 (4.2)
Weight, kg, median (SD)	22.9 (12.1)	21.9 (10.6)	22.9 (11.5)
Weight for age *z* score ≤2	35 (39)	34 (38)	29 (32)
Slept under treated net previous night	49 (54)	45 (50)	51 (57)
Prevalent parasitemia			
By microscopy^a^	0 (0)	0 (0)	1 (1.4)
By PCR	11 (12.2)	6 (6.7)	6 (6.7)

Data are no. or no. (%) of patients, unless otherwise indicated.

Abbreviations: Hb, hemoglobin; MQAS, mefloquine-artesunate; PCR, polymerase chain reaction; PCV, packed cell volume; SD, standard deviation; SPAQ, sulfadoxine-pyrimethamine plus amodiaquine.

^a^ Data are for 74 subjects in the MQAS group, 77 in the SPAQ group, and 73 in the proguanil group.

### Adherence

Doses of study drugs administered are shown in Supplementary Table 2. Adherence to visits once every 2 months was good, with most patients returning promptly at approximately 8-week intervals (Supplementary Table 3). Pill counts at the clinic suggested that all patients took both home doses of MQAS or SPAQ, although this could not be verified. Adherence to daily proguanil doses, also based on tablet counts at the clinic, was poor, with 57% of patients using <75% of daily doses. Adherence was better in children aged <10 years than in older children (Supplementary Table 4).

### Adverse Events

Severe illnesses requiring hospital admission were common in all treatment groups (0.6 episodes/person during the study period; Table 2). The most common causes for admission were vasoocclusive crisis and septicemia (Supplementary Table 6). Admissions were more frequent among female participants than male participants, in all 3 groups. Less severe illnesses treated in outpatient settings were most frequent in the proguanil group, especially among females. The protective efficacy against episodes of outpatient illness (calculated as the percentage reduction in the number of episodes), compared with the proguanil group, was 23% (95% confidence interval [CI], 0%–43%) for MQAS and 25% (95% CI, 0%–44%) for SPAQ (Table 2). The excess number of outpatient illness episodes in the proguanil group was mainly among females (interaction between sex and treatment group, *P* = .023; Table 3).

**Table 2.** Rates of Hospital Admissions and Outpatient Clinic Attendance for Illness

Variable	Hospital Admission^a^			Outpatient Clinic Attendance		
	Events, No.	Annual Rate	Adjusted RR (95% CI)^b^	Events, No.	Annual Rate	Adjusted RR (95% CI)^b^
Prophylaxis group						
Proguanil	59	0.61	1	140	1.4	1
MQAS	60	0.66	1.1 (.73–1.7)	107	1.2	0.77 (.57–1.0)
SPAQ	56	0.59	1.0 (.62–1.7)	104	1.1	0.75 (.56–1,0)
Age, y						
<5	62	0.70	1	135	1.5	1
5–9	61	0.67	0.96 (.62–1.5)	113	1.2	0.77 (.59–1.0)
10–14	38	0.61	0.92 (.52–1.6)	58	0.92	0.59 (.41–.84)
≥15	14	0.34	0.51 (.26–1.0)	45	1.1	0.65 (.40–1.1)
Sex						
Male	75	0.51	1	148	1	1
Female	100	0.73	1.5 (1.0–2.2)	203	1.5	1.5 (1.2–1.9)

Abbreviations: MQAS, mefloquine-artesunate; SPAQ, sulfadoxine-pyrimethamine plus amodiaquine.

^a^ Data are for inpatients and patients who died.

^b^ Rate ratios (RRs) and 95% confidence intervals (CIs) were estimated using Cox regression with a random effect to allow for repeat events in the same patient. Analyses were adjusted for treatment group, age, and sex.

**Table 3.** Outpatient Attendance for Illness Among Male and Female Patients, by Prophylaxis Group

Prophylaxis Group	Males				Females			
	Events, No.	Person-Years at Risk, No.	Events/Person-Year, No.	RR (95% CI)	Events, No.	Person-Years at Risk, No.	Events/Person-Year, No.	RR (95% CI)
Proguanil	47	51.29	0.9	1	93	45.35	2.1	1
SPAQ	54	51.47	1.0	1.1 (.73–1.8)	50	43.63	1.1	0.56 (.37–.85)
MQAS	47	43.59	1.1	1.2 (.74–1.9)	60	47.51	1.3	0.62 (.42–.92)

Abbreviations: CI, confidence interval; MQAS, mefloquine-artesunate; RR, rate ratio; SPAQ, sulfadoxine-pyrimethamine plus amodiaquine.

When participants were asked about side effects on day 3 after each bimonthly visit, vomiting, body pain, and abdominal pain were the most frequently reported symptoms (Table 4) and were reported more commonly by patients who received MQAS and SPAQ than by those who received proguanil. Adverse events were mostly mild in intensity (Supplementary Table 7). An adverse event during the 3 days following treatment was reported by 24% (95% CI, 19%–21%) of patients who received MQAS, 14% (95% CI, 9.6%–18%) who received SPAQ, and 5.4% (95% CI, 3.4%–17%) who received proguanil (risk ratios, 4.5 [95% CI, 3.0–6.8] for the MQAS group vs the proguanil group and 2.6 [95% CI, 1.6–4.1] for the SPAQ group vs the proguanil group). A similar proportion of male and female participants reported side effects on day 3. Among participants who received MQAS, the risk of vomiting was substantially higher for those who received MQ doses in excess of 35 mg/kg (Supplementary Figure 1).

**Table 4.** Overall and 8 Most Common Incident Adverse Events Reported, by Time of Occurrence and Prophylaxis Group

Variable	MQAS Group	SPAQ Group	Proguanil Group
On day 3 after each visit	n = 583	n = 608	n = 595
Symptom, patients, %			
Vomiting	8.9	2.0	0.0
Body pain	2.7	2.3	2.4
Abdominal pain	5.8	1.0	0.3
Weakness	2.1	4.4	0.5
Nausea	4.3	1.3	0.3
Headache	2.7	2.1	0.3
Fever	3.1	0.7	1.2
Dizziness	2.1	2.0	0.0
Any	24.0	13.8	5.4
RR (95% CI)	4.5 (3.0–6.8)	2.6 (1.6–4.1)	1
During interval between visits	n = 554	n = 591	n = 579
Symptom, patients, %			
Body pain	3.2	4.0	7.6
Fever	3.6	3.6	4.7
Cough	1.9	2.9	1.8
Abdominal pain	2.2	1.6	2.6
Headache	0.9	2.4	1.3
Other symptoms	0.8	1.3	2.5
Vomiting	1.1	0.5	0.9
Jaundice	0.5	0.2	1.0
Any	12.5	13.4	18.1
RR (95% CI)	0.68 (.51–.91)	0.74 (.55–.98)	1

Abbreviations: CI, confidence interval; MQAS, mefloquine-artesunate; RR, rate ratio; SPAQ, sulfadoxine-pyrimethamine plus amodiaquine.

When participants were asked about side effects and illness symptoms recorded in their diary in the 2 months prior to a routine clinic visit, 18% in the proguanil group reported an adverse event, compared with 13% in each of the other groups (Table 4). Females who received proguanil were more likely than males to report side effects (23% vs 14%; adverse event risk ratio, 1.6 [95% CI, 1.1–2.3]), with body pain and abdominal pain being the symptoms more commonly reported.

Seven participants died during the study (mortality rate, 25 deaths/1000 person-years). Four of these had received MQAS (a girl aged 13 years admitted with vasoocclusive crisis precipitated by sepsis who had a blood culture positive for methicillin-susceptible *Staphylococcus aureus*, a girl aged 6 years who died at home with a suspected cerebrovascular accident, a boy aged 10 years admitted with severe anemia and sepsis, and a boy aged 1 year with a febrile illness who died at home). Three deaths were in the proguanil group (a girl aged 16 years with upper gastrointestinal tract bleeding associated with disseminated intravascular coagulation, a girl aged 9 years with acute chest syndrome with sepsis, and a girl aged 12 years who died at home from an unknown cause). There were no deaths in the SPAQ group.

### Efficacy Against Malaria

Thirty-eight episodes of clinical malaria were recorded (15 inpatients and 23 outpatients). Incidence was lower in the MQAS group, with a relative protective efficacy of 61% (95% CI, 3%–84%), compared with the proguanil group. The relative efficacy of SPAQ versus that of proguanil was 36% (95% CI, −40% to 70%; Table 5). The prevalence of *P. falciparum* infection in samples taken from all participants at each scheduled clinic visit was also lower in the MQAS group than the proguanil group (odds ratio, 0.48; 95% CI, .22–1.0; Table 6. Mean hemoglobin concentration 12 months after enrollment in the trial was similar in all groups (Supplementary Table 8).

**Table 5.** Incidence of Malaria Episodes, by Prophylaxis Group

Prophylaxis Group	Participants, No.	Events, No.	Person-Years at Risk, No.	Events/Person- Year, No.	Efficacy, % (95% CI)
Proguanil	90	19	96.64	0.20	…
SPAQ	90	12	95.10	0.13	36 (−39 to 70)
MQAS	90	7	91.10	0.08	61 (2–84)

Abbreviations: CI, confidence interval; MQAS, mefloquine-artesunate; SPAQ, sulfadoxine-pyrimethamine plus amodiaquine.

**Table 6.** Prevalence of *Plasmodium falciparum* Infections, by Prophylaxis Group

Prophylaxis Group	Overall Visits, No.	Visits With *P. falciparum* Detected		OR (95% CI)
		No.	Percentage (95% CI)	
Proguanil	353	21	5.9 (3.3–8.6)	1
SPAQ	365	24	6.6 (4.3–8.9)	1.1 (.61–2.0)
MQAS	340	10	2.9 (1.2–4.6)	0.48 (.22–1.0)

Abbreviations: CI, confidence interval; MQAS, mefloquine-artesunate; OR, odds ratio; SPAQ, sulfadoxine-pyrimethamine plus amodiaquine.

## DISCUSSION

IPT with MQAS administered when patients with SCD came for routine clinic visits was well-tolerated, mild adverse events, the most common of which were vomiting and abdominal pain, did not deter patients from returning for their clinic visits. MQAS given once every 2 months was more effective in preventing malaria than daily prophylaxis with proguanil. MQAS reduced the prevalence of parasitemia at routine clinic assessments and reduced all-cause illness episodes treated as outpatients but did not reduce the burden of severe disease requiring hospital admission. Adherence to daily proguanil prophylaxis was suboptimal, and *P. falciparum* mutations associated with antifolate resistance were common. Therefore, it is likely that proguanil prophylaxis was ineffective, although the study could not include a placebo group that would have allowed this to be assessed. We did not find that treatment with SPAQ once every 2 months was more effective than proguanil in preventing malaria episodes or reducing prevalence at routine visits, although there was a reduction in episodes of outpatient illness among patients who received SPAQ. It is likely that the gap between treatments was too long (2 months) for this regimen to give a high degree of protection.

MQ can cause neurological and psychiatric side effects and should not be used in patients with psychiatric illness or epilepsy [21]. However, these side effects have not been common when MQ has been used for treatment in Asia, Latin America, and Africa [22, 23]. Vomiting is a common MQ side effect and is dose related, and its incidence was reduced by using a split dose over 3 days. Tolerability of MQ treatment and prophylaxis has been reviewed in children by Schlagenhauf et al [24] and in pregnant women by Gonsalez et al [25]. In a study of MQAS for the treatment of uncomplicated *P. falciparum* malaria undertaken in Nigeria, Agomo et al [26] reported vomiting, dizziness, headache, abdominal discomfort, weakness, and visceral pain as common adverse events, consistent with the findings of our study. Despite the occurrence of minor side effects, the majority of the patients in the MQ arm, when asked, said they would prefer to continue with the bimonthly MQAS after the end of the study, rather than revert to daily administration of proguanil.

Illnesses treated in outpatient settings and reports of illness symptoms recorded in patient diaries were more common among female participants than among male participants who took daily proguanil. Some other studies have reported that girls with SCD can have more-frequent pain episodes than boys [27]. Sex-specific differences in adverse events have also been reported for antimalarials used for prophylaxis [28]. The excess illness in the proguanil group may reflect adverse reactions to proguanil or a sex-specific increase in illness episodes associated with failure of prophylaxis.

The lack of an effect against hospital admissions in the MQAS group, despite a high efficacy against malaria, is consistent with the low prevalence of malaria in the study population. There was limited scope to demonstrate an impact on all-cause admissions, but it is possible a greater benefit would be seen in areas with more intense malaria transmission. In the 2010 malaria indicator survey in Nigeria, the national prevalence of malaria in children aged <5 years in urban areas was 23%, compared with 48% in rural areas [29].

Malaria prevention in patients with SCD using IPT with long-acting antimalarials has a number of advantages. It is recommended that stable patients should attend a clinic every 2 months, and the IPT strategy can take advantage of these routine visits. The first dose of the 3-day regimen can be administered by clinic staff or directly observed, and adherence in taking the further 2 doses is likely to be higher than that of a regimen that requires daily treatment. The cost of the MQAS regimen compares favorably with that of the proguanil regimen (Supplementary Materials). Our trial was done in an area with year-round transmission. In areas of highly seasonal transmission, chemoprevention could be limited to the transmission period, as is now recommended for children aged <5 years in the Sahel subregion [16]. In areas where seasonal malaria chemoprevention is provided, children <5 years old with SCD could receive seasonal malaria chemoprevention as an alternative to chemoprevention at SCD clinics.

Our study is larger than previous trials of malaria prophylaxis in patients with SCD, with follow-up over a longer period, but it had a number of weaknesses. We could not monitor adherence directly, our estimates of the number of daily doses used were based on pill counts at the clinic and probably overestimate actual adherence, and adherence to daily doses and return rates to clinic may be poorer outside the context of the trial. It was not feasible to blind our study, owing to difference in the number of tablets to be taken and difficulty in adequate taste masking, and this could have biased our assessment of adverse events. Reported vomiting can include spitting out a tablet or a gag reflex on administration, so data on the incidence of vomiting may overestimate the true incidence. The number of malaria events was low (the study was powered primarily to assess tolerability), and the estimates of efficacy were correspondingly imprecise. We did not investigate the pharmacokinetics of MQ used for bimonthly prophylaxis, and the incidence of malaria was too low to be able to estimate duration of protection directly, but such studies would be useful.

A high burden of severe disease was observed, with a rate of 0.6 episodes requiring admission per person per year and a mortality rate of 25 episodes per 1000 persons per year. Additional strategies are needed to reduce the burden of severe disease in patients with SCD in Africa. In the United States, an improvement in survival among patients with SCD has been attributed to introduction of pneumococcal and *Haemophilus influenzae* type b vaccination. Nigeria introduced pentavalent vaccine, which included *H. influenzae* type b vaccine, in 2012 and plans to introduce pneumococcal vaccine, and the impact of these vaccines on survival among patients with SCD needs to be evaluated. A recent survey of SCD management practices in Nigeria [30] found that few provided penicillin prophylaxis or pneumococcal vaccination for patients with SCD. All 18 clinics surveyed provided malaria prophylaxis, but our study indicates that regimens that are more effective than daily proguanil need to be used.

## Supplementary Data

Supplementary materials are available at *The Journal of Infectious Diseases* online (http://jid.oxfordjournals.org). Supplementary materials consist of data provided by the author that are published to benefit the reader. The posted materials are not copyedited. The contents of all supplementary data are the sole responsibility of the authors. Questions or messages regarding errors should be addressed to the author.

Supplementary Data
