# Supplementary material for: A Randomized Trial to Compare the Safety, Tolerability, and Effectiveness of 3 Antimalarial Regimens for the Prevention of Malaria in Nigerian Patients With Sickle Cell Disease
Source: J Infect Dis. 2015 Feb 20;212(4):617–25. doi: 10.1093/infdis/jiv093 (PMC4512609; doi:10.1093/infdis/jiv093)
Supplement: Supplementary Data [file supp_212_4_617__index.html]

A Randomized Trial to Compare the Safety, Tolerability, and Effectiveness of 3 Antimalarial Regimens for the Prevention of Malaria in Nigerian Patients With Sickle Cell Disease — A Randomized Trial to Compare the Safety, Tolerability, and Effectiveness of 3 Antimalarial Regimens for the Prevention of Malaria in Nigerian Patients With Sickle Cell Disease — Supplementary Data 

# A Randomized Trial to Compare the Safety, Tolerability, and Effectiveness of 3 Antimalarial Regimens for the Prevention of Malaria in Nigerian Patients With Sickle Cell Disease

## Supplementary Data

Supplementary Data

**Files in this Data Supplement:**

- Supplementary Data - Docx file
