# Supplementary material for: A Randomized Trial to Compare the Safety, Tolerability, and Effectiveness of 3 Antimalarial Regimens for the Prevention of Malaria in Nigerian Patients With Sickle Cell Disease
Source: J Infect Dis. 2015 Feb 20;212(4):617–25. doi: 10.1093/infdis/jiv093 (PMC4512609; doi:10.1093/infdis/jiv093)

**SUPPLEMENTARY INFORMATION**

**A randomized trial to compare the safety, tolerability and effectiveness of three antimalarial regimens for the prevention of malaria in Nigerian patients with sickle-cell disease**

Rasaq Olaosebikan, Kolade Ernest, Kalifa Bojang, Olugbenga Mokuolu, Andrea Rehman, Muna Affara, Davis Nwakanma, Jean-René Kiechel, Taofik Ogunkunle,Tope Olagunju, Rukayat Murtala, Peter Omefe,Tosin Lambe, Surajudeen Bello, Olayinka Ibrahim, Benedict Olorunsola, Ayotade Ojuawo, Brian Greenwood, Paul Milligan

**Methods**

**Laboratory methods**

**Dosage**

**Statistical methods**

**Prices of the drug regimens**

**Study site description**

**Supplementary tables:**

**Table S1:** Biochemical and haematological parameters at baseline.

**Table S2**: Dose of study drugs (based on dosage regimen, and body weight at baseline, assuming full compliance).

**Table S3**: Attendance at bimonthly clinic visits.

**Table S4**: Use of daily proguanil doses based on tablet counts at the clinic, by age group

**Table S5**: Prevalence of parasitaemia by microscopy, at scheduled clinic visits.

**Table S6**: Causes of inpatient admissions and illnesses treated as outpatients.

**Table S7**: Percentage of patients reporting mild, moderate or severe adverse events when interviewed on day 3 after each bimonthly visit.

T**able S8:** Mean Heamoglobin concentration at 12 months

**Supplementary figures:**

**Figure S1:** Incidence of reported vomiting in relation to mefloquine dose. Error bars show 95% confidence intervals.

**Figure S2:** Causes of illnesses seen at the outpatient clinic and of inpatient admission

**Methods**

***Laboratory Methods:*** A 5ml venous blood sample was taken at each 2-monthly visit for biochemical analysis (serum creatinine, urea, total protein, albumin, total bilirubin, conjugated bilirubin, alanine transaminase and aspartate transaminase) and haematology (complete blood count). Packed cell volume (PCV) was measured in the clinic by centrifugation. If malaria was suspected, a rapid diagnostic test (RDT) (First Response® Malaria Ag. (pLDH / HRP2) Combo Rapid Diagnostic Test) was done. The PCV and the malaria RDT results were made available immediately at the clinic and were used to guide patient management. Blood smears stained with Giemsa were read later by trained microscopists in the hospital laboratory and at MRC Laboratories, The Gambia. Blood spots were collected on Whatman 3MM chromatography filter paper and the dried blood spots (DBS) sent to the Medical Research Council (MRC) Unit in The Gambia for PCR analysis by RFLP. Molecular diagnosis was done on nucleic acid extracted from the dried blot spots collected at enrolment and at visits 2, 5 and 6 (due to logistic constraints we could not analyse samples from all visits). Diagnostic PCR was carried out by amplification of the multi copy ribosomal RNA gene sequence (Snounou et al. 1993, Mol Biochem Parasitol 61: 315-20; Rougemont et al., J. Microbiol 42: 5636-5643). PCR products were analysed using the QIAxcel automated capillary gel electrophoresis system (Qiagen) and fragment sizes analysed using both automated and manual scoring. Samples identified as PCR positive for Plasmodium were selected for detection of drug resistance mutations in the Pfmdr, Pfdhfr and Pfdhps genes. Nested PCR was used to amplify fragments at the three gene loci, which encompass mutations associated with pyrimethamine resistance (*dhfr*-51, 59, 108, 164), sulfadoxine resistance (*dhps*-436, 437, 540, 581) and chloroquine resistance (mdr-81) (Abdel-Muhsin et al. 2003, Parasitology 126: 391-400; Plowe et al. 1995, Am. J. Trop. Med. Hyg 52: 565 - 568).

***Dosage:*** MQAS tablets come in two strengths: artesunate 25 mg + mefloquine hydrochloride 55 mg paediatric tablets or artesunate 100 mg + mefloquine hydrochloride 220 mg adult tablets. Patients weighing 5–8 kg received one paediatric tablet per day for three days, those weighing 9–17 kg two paediatric tablets per day, those weighing 18–29 kg one adult tablet and those weighing 30 kg and above two adult tablets per day for three days, resulting in a mean dose of 28.8mg/kg over 3 days (range 19.4-44), or 9.6 mg/kg/day (range 6.5-14.7). For SPAQ, tablets containing 25mg pyrimethamine and 500 mg sulfadoxine (Micro Labs Ltd, Hour, India) and 150mg amodiaquine base [Kinepharma, Ghana] were used, (doses to the nearest half tablet at 10mg/kg/day for three days for amodiaquine and 25/1.25 mg/kg on the first day for SP). Patients received a mean total dose of AQ of 30.0mg/kg (range 20.5-37.5). Proguanil tablets (100mg as hydrochloride, Waterland Laboratories, The Netherlands) were given daily, ¼ tablet per day (<1yr of age), ½ tablet per day (1-4yrs of age), 1 tablet per day (5-8yrs), 1.5 tablets per day (9-14yrs) and 2 tablets per day (15 years and above). Tablets were crushed and mixed with water prior to administration to young children. Participants were observed for 30 minutes after drug administration; if the child vomited the dose was repeated.

***Statistical methods and sample size calculation:*** The primary endpoint of the trial was the occurrence of any adverse event. Assuming that the risk of an adverse event varies from child to child with a coefficient of variation of 0.25, if 20% of children have an adverse event in the group with the highest incidence of adverse events, a sample size of 80 per group was required to have 80% power to detect a 40% reduction (to 12%) in the occurrence of adverse events in the comparison group (Hayes R and Bennett S (1999) Int. J. Epidemiol. (1999) 28 (2): 319-326). Allowing for loss to follow up (assuming an average of 5 measurements would be obtained) a sample size of 90 was required in each group (a total of 270). Secondary endpoints included the occurrence of vomiting, adherence to the regimen, malaria incidence and incidence of other illnesses.

Incidence rates of malaria, of illnesses seen at the outpatient clinic, and of inpatient admission, were calculated as the number of events divided by total follow-up time. Patients who died, withdrew or were lost to follow-up were censored at their date of death, withdrawal or one month after the last visit date. Rate ratios were estimated using Cox regression with a random effect to allow for repeat events in the same patient. Confidence intervals for the average proportion of patients with an adverse event were calculated, allowing for grouping by patient, using a ratio estimator for the standard error. Risk ratios for the incidence of adverse events were estimated using a generalised estimating equation Poisson regression, odds ratios were calculated using random effects logistic regression. Analyses were done using Stata 12.1 (College Station, Texas).

**Prices of the regimens:**

In Ilorin a pack of 100 proguanil tablets costs from 350 to 500 Naira and two months’ supply costs from $0.32 to $0.46 for an infant, and from $2.6 to $3.7 for an adult (assuming a rate of 0.61 USD per 100Naira), compared to the cost of a single treatment of MQAS of about $1.22 (for an infant <9kg), $1.45 (9-17kg), $1.9 (18-29kg) and $3 (adult), and about 0.25$ (infant) 0.28$ (child) and 0.84$ (adult) for one treatment of SPAQ. Drugs were provided free to study participants.

**Study site*:***

This study was undertaken at the sickle cell disease clinic of the Outpatient Unit of the University of Ilorin Teaching hospital, a Federal Government-owned tertiary institution in Nigeria. The hospital, which is located in Ilorin metropolis, the capital town of Kwara state, also serves as a general hospital due to the lack of adequate infrastructure and manpower in the state government-owned secondary health facilities, and caters for patients who are referred from all areas of Kwara State and parts of the five neighbouring states of Oyo, Osun, Niger, Kogi and Ekiti. The sickle cell clinic of the hospital is run once a week and sees an average of 40 patients per clinic. Stable patients are given appointments to attend the clinic once every two to three months while patients who present with signs and symptoms of illness are given more frequent appointments. Kwara state is one of the most heterogeneous in the country and represents both in its situation and culture, a confluence of the southern and northern parts of the country. Kwara State lies in the middle belt of the country with vegetation consisting mainly of grassland and shrubs. It has dry and wet seasons typical of the Guinea savannah belt. The population of Ilorin was 847,582 in 2006 (2006 National census). Malaria is endemic, transmission occurring all year round but with an upsurge from April-June and October-December. Artemether-lumefantrine is the recommended first line treatment for malaria.

**SUPPLEMENTARY TABLES**

**Table S1:** Biochemical and haematological parameters at baseline.

| Variable | MQ -AS | SP+AQ | Proguanil |
| --- | --- | --- | --- |
| Creatinine(mmol/L),mean(sd) | 58.8(17.8) | 55.6(16.7) | 57.3(14.9) |
| Urea(mmol/L), mean (sd) | 3.0(1.2) | 2.8(1.1) | 2.8(1.0) |
| Total bilirubin(µmol/L), median (IQR) | 8.0(5, 14) | 10(6, 13) | 8(5, 14) |
| Conjugated bilirubin(µmol/L), median (IQR) | 4(2, 6) | 4(3, 7) | 4(2, 6) |
| Alanine transaminase(IU/L),median(IQR) | 8(5, 11) | 8(5, 11) | 8(4, 13) |
| Aspartate transaminase(IU/L),median(IQR) | 15(11, 26) | 17(11, 32) | 19(11, 28) |
| Total protein(G/L), mean (sd) | 68.7(6.8) | 68.9(7.9) | 69.9(8.2) |
| Albumin(G/L), mean (sd) | 36.5(6.0) | 37.4(5.0) | 37.7(5.9) |
| Haematology mean (sd): |  |  |  |
| MCV(fL) | 80.9(8.5) | 82.4(8.1) | 81.3(7.9) |
| MCHC(g/dl) | 31.0(2.3) | 30.8(4.0) | 31.2(3.9) |
| MCH(pg) | 25.1(3.3) | 25.4(4.3) | 25.3(3.9) |
| Platelets(×10^9^/L) | 373.8(144.4) | 405.5(180.6) | 361.9(173.7) |
| White blood cells (×10^9^ cells/L) | 12.0(5.2) | 11.9(5.0) | 11.3(5.6) |

**Table S2**: Dose of study drugs (based on dosage regimen, and body weight at baseline, assuming full compliance).

|  | MQAS | SPAQ | Proguanil |
| --- | --- | --- | --- |
| Mean weight in kg (range) | 23.0 (7.6-56) | 22.0 (6.0-60) | 23.0 (8.1-56) |
| Mefloquine mg/kg total over 3 days (range) | 28.8 (19.4-44.0) |  |  |
| Sulfadoxine mg/kg per dose (range) |  | 25.8 (17.9-41.7) |  |
| Pyrimethamine mg/kg per dose (range) |  | 1.29 (0.89-2.08) |  |
| Amodiaquine mg/kg total over 3 days (range) |  | 30.0 (20.5-37.5) |  |
| Proguanil mg/kg/day (range) |  |  | 5.1 (2.8-12.5) |

**Table S3**: Attendance at bimonthly clinic visits.

|  | MQ-AS | SP+AQ | Proguanil |
| --- | --- | --- | --- |
| Attendance at bimonthly clinic visits:  % patients that attended 6 times | 89% | 93% | 91% |
| % patients that attended 7 times | 77% | 83% | 67% |
| Percentiles of the no. of weeks between clinic visits:  10% | 7.7 | 7.7 | 7.0 |
| 50% | 8.0 | 8.0 | 8.0 |
| 90% | 9.0 | 9.0 | 15 |

**Table S4**: Use of daily proguanil doses based on tablet counts at the clinic, by age group.

|  | <10yrs | ≥10yrs | Total | Total* |
| --- | --- | --- | --- | --- |
| No. of patients | 55 | 35 | 90 | 90 |
| median % daily doses used | 86% | 82% | 85% | 73% |
| % of patients that used at least 90% of daily doses | 36% | 14% | 28% | 8.8% |
| % of patients that used at least 80% of daily doses | 75% | 66% | 71% | 18% |
| % of patients that used less than 75% of daily doses | 15% | 29% | 20% | 57% |

^*^Mean adherence over 14 months, assuming zero adherence for periods without a supply of tablets for patients who stopped attending clinic before the end of the study.

**Table S5**: Prevalence of parasitaemia by microscopy, at scheduled clinic visits.

|  | MQ-AS | SP+AQ | Proguanil | No. sampled |
| --- | --- | --- | --- | --- |
| Enrolment | 0% | 0% | 1.40% | 224 |
| Visit 2 | 2.2% | 11.4% | 7.1% | 131 |
| Visit 3 | 1.8% | 1.5% | 4.6% | 187 |
| Visit 4 | 2.4% | 4.3% | 13.3% | 133 |
| Visit 5 | 1.6% | 8.7% | 4.3% | 203 |
| Visit 6 | 4.8% | 8.8% | 3.2% | 192 |
| Visit 7 | 4.3% | 5.5% | 5.8% | 212 |
| Mean (visits 2-7) | 2.9% | 6.6% | 5.9% | 1282 |
| Odds ratio (95%CI) | 0.48 (0.22,1.0) | 1.1 (0.61,2.0) | 1 |  |

**Table S6**: Causes of inpatient admissions and outpatient illnesses.

| ` | Inpatients | |  |  | Outpatients | |  |  |
| --- | --- | --- | --- | --- | --- | --- | --- | --- |
| Variable | MQAS | SPAQ | Proguanil | Total | MQAS | SPAQ | Proguanil | Total |
|  | N=58 | N=53 | N=55 | 166 | N=105 | N=101 | N=137 | 343 |
| Vaso-occlusive crisis | 58.6% | 69.8% | 67.3% | 65.1% | 49.5% | 33.7% | 46.7% | 43.7% |
| Septicaemia | 27.6% | 22.6% | 30.9% | 27.1% | 5.7% | 5.0% | 9.5% | 7.0% |
| Osteomyelitis | 10.3% | 11.3% | 14.5% | 12.0% | 2.9% | 0.0% | 0.7% | 1.2% |
| Severe anaemia | 10.3% | 9.4% | 14.5% | 11.4% | 1.9% | 0.0% | 1.5% | 1.2% |
| Haemolytic crisis | 12.1% | 9.4% | 10.9% | 10.8% | 1.0% | 0.0% | 2.9% | 1.5% |
| Malaria | 8.6% | 5.7% | 12.7% | 9.0% | 1.9% | 8.9% | 8.8% | 6.7% |
| Pneumonia | 10.3% | 3.8% | 5.5% | 6.6% | 3.8% | 4.0% | 2.9% | 3.5% |
| Skin/soft tissue infection | 5.2% | 3.8% | 9.1% | 6.0% | 7.6% | 9.9% | 9.5% | 9.0% |
| Gastroenteritis | 1.7% | 1.9% | 3.6% | 2.4% | 1.0% | 3.0% | 1.5% | 1.7% |
| Pharyngitis | 3.4% | 1.9% | 0.0% | 1.8% | 9.5% | 6.9% | 8.0% | 8.2% |
| Urinary tract infection | 0.0% | 3.8% | 0.0% | 1.2% | 5.7% | 1.0% | 2.9% | 3.2% |
| URTI | 3.4% | 0.0% | 0.0% | 1.2% | 11.4% | 11.9% | 7.3% | 9.9% |
| Others | 8.6% | 17.0% | 3.6% | 9.6% | 14.3% | 23.8% | 20.4% | 19.5% |
|  |  |  |  |  |  |  |  |  |
| % of patients with more than one diagnosis | 41% | 47% | 51% | 46% | 15% | 7.9% | 20% | 15% |
| No. transfused | 10 | 11 | 13 | 34 | 0 | 0 | 0 | 0 |
| Gender F:M | 36:22 | 28:25 | 30:25 | 94:72 | 59:46 | 49:52 | 92:45 | 200:143 |
| Mean age in years  (range) | 6.6  1-17 | 8.0  1-21 | 7.7  0.7-21 | 7.3  0.7-21 | 6.7  1-17 | 7.0  0.6-21 | 7.9  0.7-18 | 7.3  0.6-21 |

**Table S7**: Percentage of patients reporting mild, moderate or severe adverse events when interviewed on day 3 after each bimonthly visit.

|  | Mild |  |  | Moderate | |  | Severe |  |  |
| --- | --- | --- | --- | --- | --- | --- | --- | --- | --- |
| Visit | MQAS | SPAQ | Proguanil | MQAS | SPAQ | Proguanil | MQAS | SPAQ | Proguanil |
| 1 | 34.4% | 20.0% | 8.9% | 4.4% | 1.1% | 0.0% | 1.1% | 1.1% | 0.0% |
| 2 | 21.6% | 18.0% | 13.5% | 3.4% | 0.0% | 1.1% | 0.0% | 0.0% | 0.0% |
| 3 | 23.8% | 13.5% | 5.7% | 0.0% | 1.1% | 0.0% | 0.0% | 0.0% | 0.0% |
| 4 | 20.7% | 7.9% | 1.2% | 1.2% | 2.2% | 0.0% | 0.0% | 1.1% | 0.0% |
| 5 | 24.4% | 15.9% | 1.2% | 0.0% | 1.1% | 1.2% | 0.0% | 0.0% | 1.2% |
| 6 | 27.5% | 6.0% | 2.5% | 0.0% | 3.6% | 1.2% | 1.3% | 0.0% | 0.0% |
| 7 | 15.6% | 13.9% | 3.8% | 2.6% | 2.5% | 0.0% | 0.0% | 0.0% | 0.0% |
| Mean | 24.2% | 13.7% | 5.4% | 1.7% | 1.6% | 0.5% | 0.3% | 0.3% | 0.2% |

T**able S8: Mean Heamoglobin concentration at 12 months**

|  | Mean (95%CI) |
| --- | --- |
| Proguanil | 7.76 (7.45-8.07) n=81 |
| SPAQ | 7.62 (7.33- 7.91) n=80 |
| MQAS | 7.49 (7.22- 7.76) n=77 |

(one-way ANOVA p-value=0.43)

**SUPPLEMENTARY FIGURES**

**Figure S1**: Incidence of reported vomiting in relation to mefloquine dose. Error bars show 95% confidence intervals.


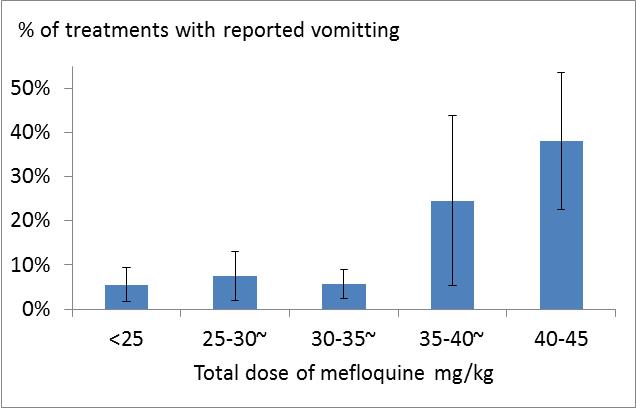


**Figure S2:** Causes of outpatient consultations and inpatient admission


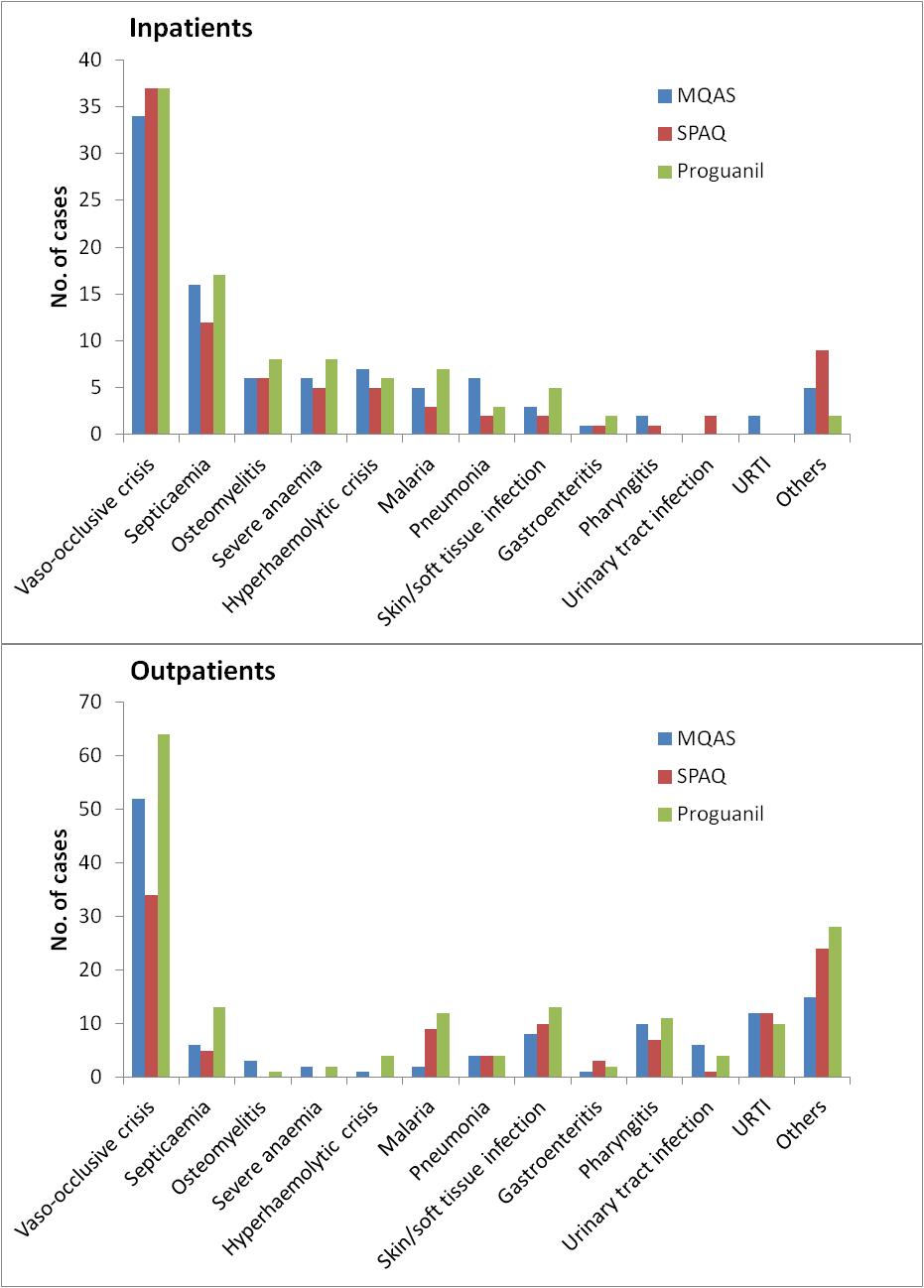

Supplement: Supplementary Data [file supp_jiv093_jiv093supp.docx]
